# Workload of diagnostic radiologists in the foreseeable future based on recent scientific advances: growth expectations and role of artificial intelligence

**DOI:** 10.1186/s13244-021-01031-4

**Published:** 2021-06-29

**Authors:** Thomas C. Kwee, Robert M. Kwee

**Affiliations:** 1 Medical Imaging Center, Departments of Radiology, Nuclear Medicine and Molecular Imaging, University Medical Center Groningen, University of Groningen, Hanzeplein 1, P.O. Box 30.001, 9700 RB Groningen, The Netherlands; 2 Department of Radiology, Zuyderland Medical Center, Heerlen, Sittard-Geleen, The Netherlands

**Keywords:** Artificial intelligence, Radiology, Radiologists, Research, Workload

## Abstract

**Objective:**

To determine the anticipated contribution of recently published medical imaging literature, including artificial intelligence (AI), on the workload of diagnostic radiologists.

**Methods:**

This study included a random sample of 440 medical imaging studies published in 2019. The direct contribution of each study to patient care and its effect on the workload of diagnostic radiologists (i.e., number of examinations performed per time unit) was assessed. Separate analyses were done for an academic tertiary care center and a non-academic general teaching hospital.

**Results:**

In the academic tertiary care center setting, 65.0% (286/440) of studies could directly contribute to patient care, of which 48.3% (138/286) would increase workload, 46.2% (132/286) would not change workload, 4.5% (13/286) would decrease workload, and 1.0% (3/286) had an unclear effect on workload. In the non-academic general teaching hospital setting, 63.0% (277/240) of studies could directly contribute to patient care, of which 48.7% (135/277) would increase workload, 46.2% (128/277) would not change workload, 4.3% (12/277) would decrease workload, and 0.7% (2/277) had an unclear effect on workload. Studies with AI as primary research area were significantly associated with an increased workload (*p* < 0.001), with an odds ratio (OR) of 10.64 (95% confidence interval (CI) 3.25–34.80) in the academic tertiary care center setting and an OR of 10.45 (95% CI 3.19–34.21) in the non-academic general teaching hospital setting.

**Conclusions:**

Recently published medical imaging studies often add value to radiological patient care. However, they likely increase the overall workload of diagnostic radiologists, and this particularly applies to AI studies.

**Supplementary Information:**

The online version contains supplementary material available at 10.1186/s13244-021-01031-4.

## Key points

- In an academic tertiary care center, 65.0% of recently published medical imaging studies could directly contribute to patient care, of which 48.3% would increase and 4.5% would decrease workload.
- In a non-academic general teaching hospital, 63.0% of recently published medical imaging studies could directly contribute to patient care, of which 48.7% would increase and 4.3% would decrease workload.
- Recently published medical imaging studies with AI as primary research area are significantly associated with an increased workload.

## Introduction

The workload of radiologists (i.e., the product of the number and complexity of examinations performed per time unit) has increased considerably over the past decades. This is largely due to the growth in the number of cross-sectional imaging examinations (particularly CT and MRI), increased complexity of image interpretation because of the acquisition of larger datasets, and declining imaging reimbursements [1–4]. The latter forces radiology practices to increase productivity to maintain income levels, while limiting their financial possibilities to employ new staff. Consequently, the overall workload per radiologist has considerably increased over the past years. Not surprisingly, burnout is recognized as an increasingly important problem among radiologists [5, 6]. Work overload may also compromise the quality and safety of patient care that can be provided by radiologists [7–9].

There are many factors that influence the utilization of medical imaging [10]. Scientific evidence can be considered as perhaps the most important driver, because research-based advances shape the way how radiology practice is performed in the future. Currently, however, it is unclear how recently published literature contributes to the workload in the field of radiology. This information would be important to radiologists and other stakeholders (including healthcare systems and governmental bodies) who aim to maintain a radiology workforce that is sufficiently large to meet clinical demands at high standards.

Meanwhile, there are also voices that speculate that artificial intelligence (AI) is expected to speed up scan time, make more accurate diagnoses, and ease the workload of radiologists [11]. Although the speculation that AI will decrease workload has not yet been supported by evidence, it can already have an important impact on political and strategic decisions. Based on this speculation, policy makers may already decide not to increase or even to limit the number of residents that can be enrolled into radiology training programs, may limit financial resources to employ (new) radiologists, and may further cut reimbursements for imaging procedures. This may have detrimental effects that cannot be rapidly reversed when the workload of radiologists actually proves to keep on increasing. Interestingly, in a recent survey among European Society of Radiology (ESR) members, 74.7% (504/675) expected an impact of AI on total reporting workload, with 50.8% (256/504) of them expecting a reduced reporting workload and 49.2% (248/504) expecting the opposite scenario [12]. Since the effect of AI on future workload remains unclear, and associated practical consequences are very relevant, it is crucial to fill in this knowledge gap. It is hypothesized that most recent scientific evidence increases workload in radiology practice, while there is no net effect of AI on radiologists’ workload based on the recent ESR survey results [12].

The purpose of this study was therefore to determine the anticipated contribution of recently published medical imaging literature on the workload of diagnostic radiologists.

## Materials and methods

### Study design

This study used literature data and did not require ethical review board approval or patient consent. Studies that were published in any of the 20 highly ranked clinical imaging journals and 20 highly ranked general medicine and clinical specialty journals (according to impact factor in the Journal Citation reports [13], that are listed in Table 1, were considered for inclusion in this study. All studies that were published in these 40 clinical journals in 2019 were retrieved from PubMed/Medline using the search term that is displayed in Additional file 1: Appendix 1. Using the random number generator in Microsoft Excel (Microsoft Corporation), a random sample of 1,000 studies was drawn from the studies that were retrieved by the PubMed/Medline search. These 1,000 studies were reviewed for eligibility by consensus of two radiologists (T.C.K. & R.M.K.). Studies were included if they concerned diagnostic imaging studies. Only original studies, systematic reviews and meta-analyses, were included. Case reports, conference proceedings, editorials, errata, guidelines/society statements, studies dealing with interventional radiology, letters to the editor, meeting summaries, non-systematic reviews, and studies clearly not related to medical imaging, were excluded. Studies dealing with imaging procedures that are not performed by radiologists in the authors’ institutions, such as cardiac ultrasonography or endoscopic ultrasonography, were also excluded. Studies were excluded if clearly ineligible based on title and abstract; otherwise, the full text version was evaluated.

**Table 1:** Overview of 20 clinical imaging journals and 20 general medicine and clinical specialty journals whose studies were potentially eligible for inclusion

Imaging journals	General medicine and clinical specialty journals
JACC: Cardiovascular Imaging	New England Journal of Medicine
Radiology	Lancet
Journal of Nuclear Medicine	Journal of the American Medical Association
European Journal of Nuclear Medicine and Molecular Imaging	Lancet Oncology
Clinical Nuclear Medicine	Journal of Clinical Oncology
Journal of Cardiovascular Magnetic Resonance	BMJ
Investigative Radiology	Lancet Neurology
European Heart Journal—Cardiovascular Imaging	Lancet Diabetes & Endocrinology
Journal of the American College of Radiology	Lancet Respiratory Medicine
European Radiology	JAMA Oncology
Journal of Magnetic Resonance Imaging	Lancet Infectious Diseases
Insights into Imaging	Circulation
American Journal of Neuroradiology	European Heart Journal
Journal of Nuclear Cardiology	Annals of Internal Medicine
Quantitative Imaging in Medicine and Surgery	Journal of the American College of Cardiology
Clinical Neuroradiology	Gut
Korean Journal of Radiology	European Urology
Journal of Vascular and Interventional Radiology	JAMA Internal Medicine
American Journal of Roentgenology	Annals of Oncology
European Journal of Radiology	Blood

### Study analysis

One radiologist affiliated to an academic tertiary care center in the north of the Netherlands (T.C.K., radiologist 1), and another radiologist affiliated to a non-academic general teaching hospital in the south of the Netherlands (R.M.K., radiologist 2), independently reviewed the full text of each included study. Radiologist 1 had 5 years of post-residency clinical radiology experience and radiologist 2 had 6 years of post-residency clinical radiology experience, and both radiologists had 15 years of experience in critically appraising scientific literature in radiology. The radiology departments in both institutions, which are among the largest in The Netherlands, are regularly audited by the Radiological Society of the Netherlands to fulfill quality standards of radiological care [14]. Both radiologists assessed whether the results of each individual study could directly contribute to patient care in their radiology practice (i.e., whether the results may be directly implemented in practice for the purpose of enhancing patient care). If this was the case, this contribution was then categorized as concerning:

1. a completely new imaging application in their radiology practice.
2. another type of imaging as an alternative for an existing imaging application in their radiology practice.
3. an elaboration of on an existing imaging application in their radiology practice.

For contributions in category (2), it was qualitatively determined if the acquisition, post-processing, and interpretation times of the alternative type of imaging would be shorter than, as long as, or longer than that of the existing imaging application, or whether this remained unclear. Similarly, for contributions in category (3), it was qualitatively determined if the acquisition, post-processing, and interpretation times of the existing imaging application would decrease, remain stable, increase, or whether this remained unclear. Note that acquisition time refers to the time required to obtain images of the human body with a certain imaging modality, post-processing time refers to the time required for the manipulation of medical images to derive additional qualitative or quantitative data [15], and interpretation time refers to the time required to analyze and interpret the acquired and post-processed imaging data. All studies in category (1) and any study in categories (2) or (3) with a net increase in acquisition, post-processing, and/or interpretation time were considered to increase workload. Any study in categories (2) or (3) with a net decrease in acquisition, post-processing, and/or interpretation time was considered to decrease workload. Any study in categories (2) or (3) without a net change in acquisition, post-processing, and/or interpretation time was considered to have no effect on workload. All other studies were considered to have an unclear impact on workload. Study examples are shown in Tables 2 and 3 [16–26].

**Table 2:** Examples of studies with a direct contribution to patient care^a,b^

References	Primary research area	Description study	Category	Workload
[16]	Neuroradiology	*Study purpose:* “To assess diagnostic accuracy of MR neurography in the differential diagnosis of amyotrophic lateral sclerosis (ALS) and multifocal motor neuropathy (MMN)” *Study conclusion:* “MR neurography is an accurate method for assisting in the differential diagnosis of ALS and MMN”	Completely new imaging application	Increases (new imaging application)
[17]	Chest	*Study purpose:* “This study analyzed phantom and human chronic obstructive pulmonary disease (COPD) data to test the hypothesis that ultra-high-resolution computed tomography (U-HRCT) can accurately measure peripheral airways that are difficult to measure with conventional CT” *Study conclusion:* “U-HRCT enables accurate and direct evaluation of peripheral airways 1–2 mm in diameter. The 6th generation airways are commonly < 2 mm in diameter, and the sum-LA can be a useful CT biomarker that reflects airflow limitation in COPD”	Another type of imaging as an alternative for an existing imaging application	Increases (longer interpretation time than conventional HRCT)
[18]	Nuclear medicine	*Study purpose:* “The aim of this study was to investigate the diagnostic performance of whole-body [C]acetate PET/CT in less aggressive or indolent lymphomas, wherein [F]FDG PET/CT would exhibit limited sensitivity” *Study conclusion:* “[C]acetate PET/CT exhibited greater sensitivity than [F]FDG PET/CT for lesion detection in patients with less aggressive or indolent lymphomas, thus promising applicability as a physiological tracer in the study of such lesions”	Another type of imaging as an alternative for an existing imaging application	No change
[19]	AI	*Study purpose:* “Focal pattern in multiple myeloma (MM) seems to be related to poorer survival and differentiation from diffuse to focal pattern on CT has inter-reader variability. We postulated that a radiomic approach could help radiologists in differentiating diffuse from focal patterns on CT” *Study conclusion:* “A radiomics approach improves radiological evaluation of focal and diffuse pattern of MM on CT”	Elaboration of an existing imaging application	Increases (longer post-processing and interpretation time)
[20]	Musculoskeletal	*Study purpose:* “To assess how many and which CT reformats of long bone non-unions should be analyzed to best approximate the analysis of a larger number of CT reformats obtained in the three orthogonal planes” *Study conclusion:* “Semi-quantitative analysis of the two paramedian sagittal and coronal CT reformats is an acceptable alternative to the analysis of more numerous reformats”	Elaboration of an existing imaging application	Decreases (shorter interpretation time)
[21]	Cardiac	*Study purpose:* “To investigate the clinical utility of our newly developed contrast enhancement optimizer (CEO) software for coronary CT angiography (CCTA)” *Study conclusion:* “The use of our CEO for CCTA studies yielded optimal aortic contrast enhancement in significantly more patients than the standard protocol based on the body weight”	Elaboration of an existing imaging application	No change

^a^Based on the applicability of the methods, results, interpretations, and conclusions, as described in each study, to the patient spectrum and radiology practice in the institutions of each of the two observers. Study quality was not a factor that influenced this decision

^b^The examples shown in this table study could directly contribute to patient care in the radiology practices of both observers 1 and 2

**Table 3:** Examples of studies without a direct contribution to patient care^a,b^

References	Primary research area	Description study
[22]	Nuclear medicine	*Study purpose:* “The aim of this study is to measure acute changes in NaF uptake in human bone due to exercise-induced loading” *Study conclusion:* “Bone loading induces an acute response in bone physiology as quantified by [18F]NaF PET kinetics. Dynamic imaging after bone loading using [18F]NaF PET is a promising diagnostic tool in bone physiology and imaging of biomechanics”
[23]	Magnetic resonance	*Study purpose:* “To qualitatively and quantitatively compare the image quality between single-shot echo-planar (SS-EPI) and multi-shot echo-planar (IMS-EPI) diffusion-weighted imaging (DWI) in female pelvis” *Study conclusion:* “IMS-EPI showed better image quality with lower geometric distortion without affecting the quantification of apparent diffusion coefficient, though the signal-to-noise ratio and contrast-to-noise ratio decreased due to post-processing limitations”
[24]	Breast	*Study purpose:* “To develop a fast three-dimensional method for simultaneous T1 and T2 quantification for breast imaging by using MR fingerprinting” *Study conclusion:* “A method was developed for breast imaging by using the MR fingerprinting technique, which allows simultaneous and volumetric quantification of T1 and T2 relaxation times for breast tissues”
[25]	Gastrointestinal–abdominal	*Study purpose:* “To compare patient acceptability and burden of magnetic resonance enterography (MRE) and ultrasound (US) to each other, and to other enteric investigations, particularly colonoscopy.” *Study conclusion:* “MRE and US are well tolerated. Although MRE generates greater burden, longer recovery and is less preferred than US, it is more acceptable than colonoscopy. Patients, however, place greater emphasis on diagnostic accuracy than burden”
[26]	Urogenital	*Study purpose:* “The objectives of this study were to assess whether the accuracy of urologists in identifying the presence of clinically significant cancer based on a standardized multiparametric MRI set could be improved by completion of a 2-d training course” *Study conclusion:* “Whilst we require expert radiologists to report prostate MRI, this study has demonstrated that identification of clinically significant cancer on prostate MRI by urologists is improved following exposure to a 2-d teaching course. These results would support efforts to integrate prostate MRI teaching courses into the training of urologists managing patients with prostate cancer”

^a^Based on the applicability of the methods, results, interpretations, and conclusions, as described in each study, to the patient spectrum and radiology practice in the institutions of each of the two observers. Study quality was not a factor that influenced this decision

^b^The examples shown in this table study could not directly contribute to patient care in the radiology practices of both observers 1 and 2

### Statistical analysis

Separate analyses were done for the previously mentioned academic tertiary care center and non-academic general teaching hospital settings. The proportions of studies that could directly contribute to patient care, and those concerning (1) a completely new imaging application, (2) another type of imaging as an alternative for an existing imaging application, and (3) an elaboration of on an existing imaging application were calculated. Subsequently, the proportions of studies that would increase workload, decrease workload, have no effect on workload, and with an unclear impact on workload were calculated. Logistic regression analyses were performed to determine the association of increased workload with a study’s primary research area (AI, breast, cardiac, chest, computed tomography, contrast media, emergency, experimental, gastrointestinal–abdominal, head–neck, magnetic resonance, multisystem, musculoskeletal, neuroradiology, nuclear medicine, oncology, pediatric, ultrasonography, urogenital, or vascular) and impact factor of the journal in which the study was published (according to the 2019 Journal Citation reports (13)). Studies that would increase workload were coded as “1” and studies that would not change or decrease workload were calculated as “0” for this purpose. The largest category was used as reference for the variable “study’s research area.” *p* values less than 0.05 were considered statistically significant. Statistical analyses were executed using MedCalc version 17.2 Software (MedCalc).

## Results

### Eligible studies

**Fig. 1:**
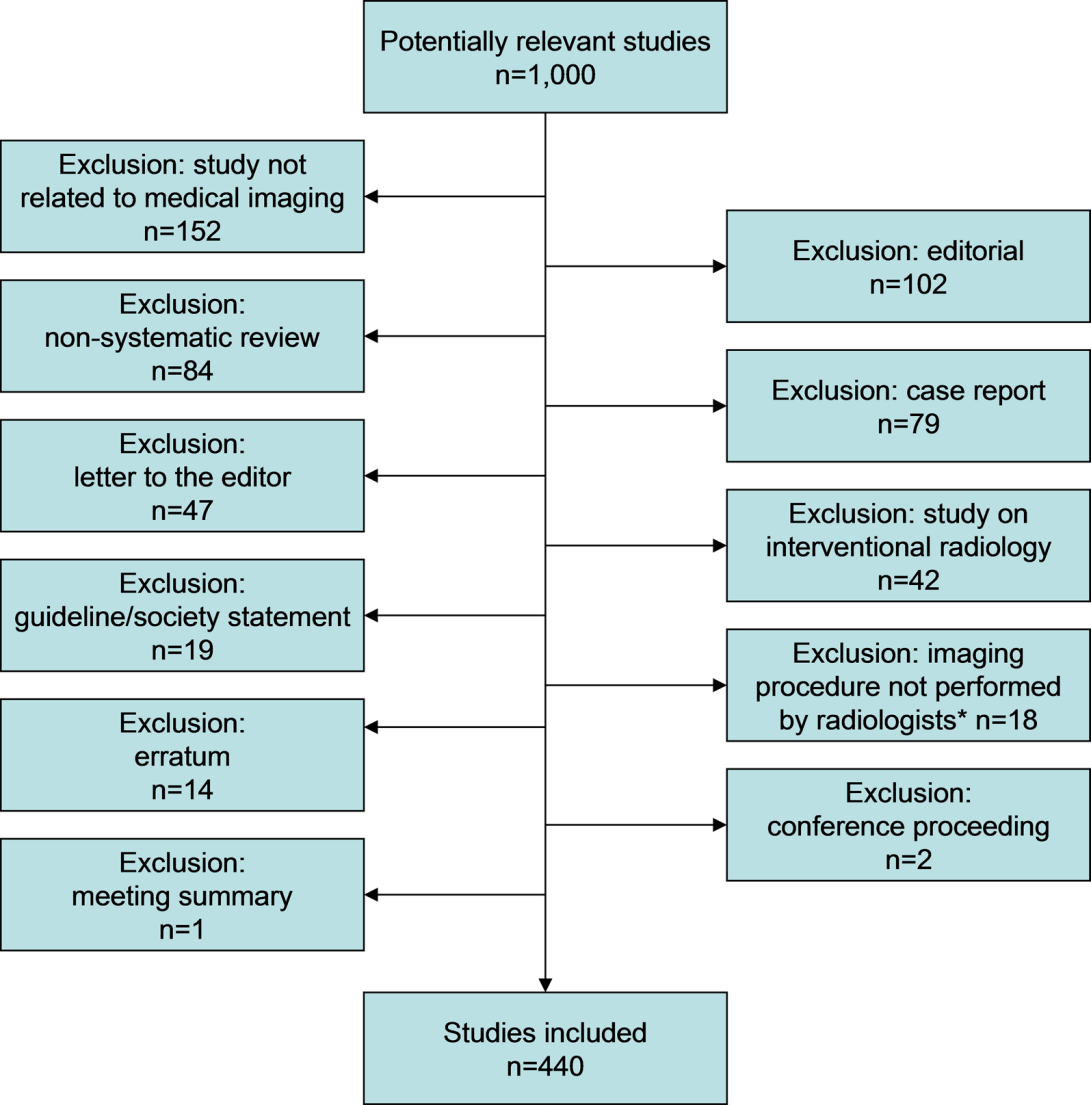
Flow diagram of the study selection process. *Notes*:*in the authors’ institutions

Of the 1000 studies that were evaluated, 560 studies were excluded (Fig. 1). Finally, 440 studies remained for inclusion (Additional file 1: Appendix 2).

### Academic tertiary care center setting

**Fig. 2:**
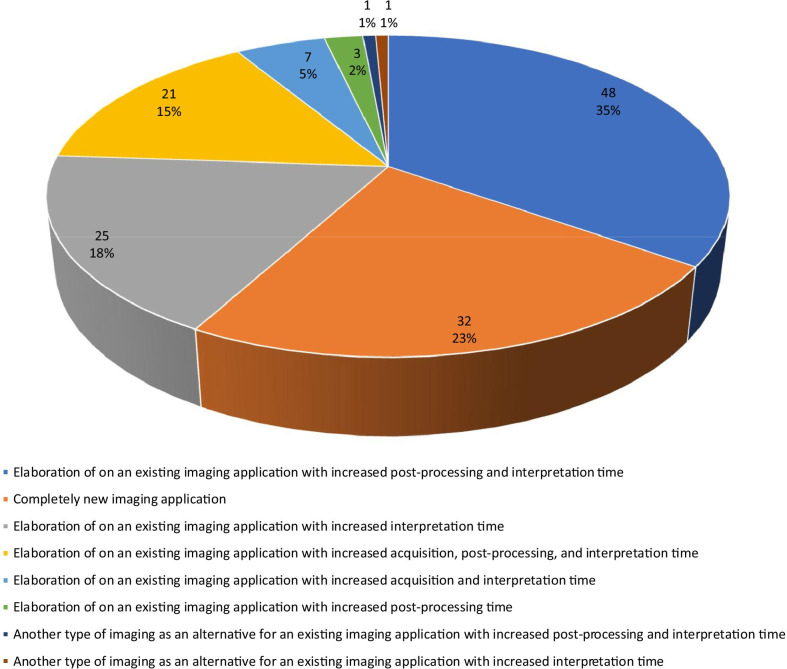
Causes of increased workload in an academic tertiary care center

65.9% (286/440) of studies could directly contribute to patient care, of which 84.6% (242/286) concerned an elaboration of an existing imaging application, 11.2% (32/286) concerned a new imaging application, and 4.2% (12/286) concerned another type of imaging as an alternative for an existing imaging application. Of the 286 studies that could directly contribute to patient care, 48.3% (138/286) would increase workload, 46.2% (132/286) would not change workload, 4.5% (13/286) would decrease workload, and 1.0% (3/286) had an unclear effect on workload. Causes of increased workload are detailed in Fig. 2. The main cause of increased workload was an increase in interpretation time of an existing imaging application (74.6%, 103/138), often combined with an increase in post-processing time and acquisition time. The second main cause of increased workload was the introduction of a completely new imaging application (23.2%, 32/138).

On univariate analysis, both AI as research area and a lower impact factor of the journal in which the study was published were significantly associated with an increased workload (Table 4). On multivariate analysis, only AI as primary research area remained significantly associated with an increased workload (*p* < 0.001), with an odds ratio (OR) of 10.64 (95% confidence interval (CI) 3.25–34.80) (Table 4). Of 38 AI studies with a potential direct contribution to patient care, 33 (86.5%) would increase workload (of which 32 due to increased post-processing and interpretation time and 1 due to increased post-processing time), 3 (7.9%) would decrease workload, 1 (2.6%) would not change workload, and 1 (2.6%) had an unclear effect on workload.

**Table 4:** Logistic regression analyses on the association of increased workload with a study’s research area and impact factor of the journal in which the study was published, for an academic tertiary care center

Variable	Univariate analysis			Multivariate analysis			
	OR	95% CI	*p* value	OR		95% CI	*p* value
Study’s research area^a^	11.79^b^	3.64–38.28^b^	< 0.001^b^	10.64^b^		3.25–34.80^b^	< 0.001^b^
Impact factor of the journal in which the study was published^c^	0.93^d^	0.86–1.00^d^	0.020	0.93^d^		0.85–1.02^d^	0.110

CI: confidence interval, OR: odds ratio

^a^Based on 286 studies with the following primary research areas: artificial intelligence (n = 38), breast (n = 16), cardiac (n = 32), chest (n = 20), computed tomography (n = 4), emergency (n = 1), gastrointestinal–abdominal (n = 21), head–neck (n = 6), magnetic resonance (n = 3), multisystem (n = 1), musculoskeletal (n = 18), neuroradiology (n = 46), nuclear medicine (n = 51), oncology (n = 2), pediatric (n = 2), ultrasonography (n = 2), urogenital (n = 17), and vascular (n = 6)

^b^Studies with artificial intelligence as research area were significantly associated with increased workload

^c^Based on 26 individual journals with a median impact factor of 5.061 (range: 2.687–33.752)

^d^Per unit increase in impact factor

### Non-academic general teaching hospital setting

**Fig. 3:**
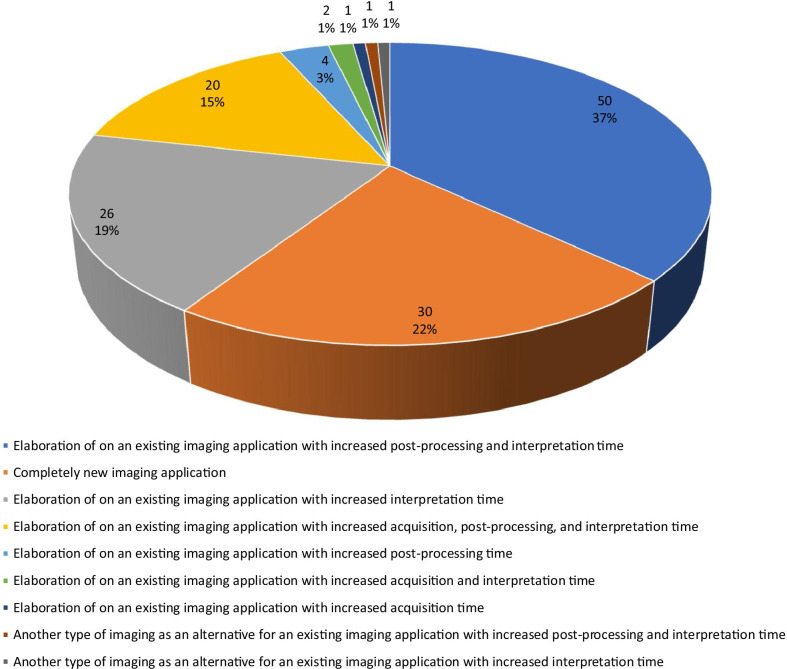
Causes of increased workload in a non-academic general teaching hospital

63.0% (277/440) of studies could directly contribute to patient care, of which 84.8% (235/277) concerned an elaboration of on an existing imaging application, 10.8% (30/277) concerned a new imaging application, and 4.3% (12/277) concerned another type of imaging as an alternative for an existing imaging application. Of the 277 studies that could directly contribute to patient care, 48.7% (135/277) would increase workload, 46.2% (128/277) would not change workload, 4.3% (12/277) would decrease workload, and for 0.7% (2/277) the effect on workload was unclear. Causes of increased workload are detailed in Fig. 3. The main cause of increased workload was an increase in interpretation time of an existing imaging application (74.1%, 100/135), often combined with an increase in post-processing time and acquisition time. The second main cause of increased workload was the introduction of a completely new imaging application (22.2%, 30/135).

On both univariate and multivariate analyses, only AI as primary research area was significantly associated with an increased workload (*p* < 0.001), with a multivariate OR of 10.45 (95% CI 3.19–34.21) (Table 5). Of 37 AI studies with a potential direct contribution to patient care, 32 (86.8%) would increase workload (of which 29 due to increased post-processing and interpretation time, 2 due to increased post-processing time, and 1 due to increased interpretation time), 3 (8.1%) would decrease workload, 1 (2.7%) would not change workload, and for 1 (2.7%) the effect on workload was unclear.

**Table 5:** Logistic regression analyses on the association of increased workload with a study’s research area and impact factor of the journal in which the study was published, for a non-academic general teaching hospital

Variable	Univariate analysis			Multivariate analysis			
	OR	95% CI	*p* value	OR		95% CI	*p* value
Study’s research area^a^	11.05^b^	3.39–36.01^b^	< 0.001^b^	10.45^b^		3.19–34.21^b^	< 0.001^b^
Impact factor of the journal in which the study was published^c^	0.94^d^	0.87–1.01^d^	0.065	0.950^d^		0.87–1.04^d^	0.268

CI: confidence interval, OR: odds ratio

^a^Based on 277 studies with the following primary research areas: artificial intelligence (n = 37), breast (n = 16), cardiac (n = 32), chest (n = 18), computed tomography (n = 4), emergency (n = 1), gastrointestinal–abdominal (n = 20), head–neck (n = 6), magnetic resonance (n = 3), multisystem (n = 1), musculoskeletal (n = 18), neuroradiology (n = 44), nuclear medicine (n = 50), oncology (n = 1), pediatric (n = 2), ultrasonography (n = 2), urogenital (n = 17), and vascular (n = 5)

^b^Studies with artificial intelligence as research area were significantly associated with increased workload

^c^Based on 25 individual journals with a median impact factor of 4.966 (range 2.687–33.752)

^d^Per unit increase in impact factor

## Discussion

The results of this study show that > 60% of recent scientific evidence in the medical imaging field may be directly implemented in diagnostic radiology practice for the purpose of enhancing patient care. However, nearly 50% of these studies would increase workload, while less than 5% would decrease workload. Therefore, the projected overall workload of diagnostic radiologists is expected to rise. Increased workload was due to an increase in interpretation time of an existing imaging application in nearly 75% (often combined with an increase in post-processing time and acquisition time) and due to the introduction of a completely new imaging application in 20–25%. Interestingly, studies with AI as primary research area were significantly associated with an increased workload; > 86% of AI studies increased workload, of which the far majority due to an increase in both post-processing and interpretation time.

Over the past decades, the workload of radiologists has already risen considerably [1–4], with a concomitant increase in the number of studies reporting high rates of burnout among radiology staff [5, 6, 27]. Implementing recent scientific innovations and advances in knowledge in diagnostic radiology practice may benefit patient care, but, as demonstrated by the present study, further increases workload. Maintaining the same radiology workforce while increasing the intensity of work (i.e., number of procedures per time unit) and/or extending working hours has been a common phenomenon among radiology practices in the past years [28–30]. However, this strategy is not a sustainable solution considering the fact that medical imaging keeps on evolving at a rapid pace, which continuously adds to the net workload of radiologists. Without any intervention, the continuous addition of workload that aims to improve patient care will eventually turn into work overload that may jeopardize the quality and safety of patient care [7–9]. AI is regarded as a potential solution to increase efficiency and ease the workload of radiologists [11, 31]. However, the present study suggests that most current AI applications in medical imaging have the opposite effect, because they commonly require additional post-processing and interpretation time rather than being seamlessly integrated in the workflow and taking over tasks of the diagnostic radiologist. Therefore, we believe that there is currently no scientific basis for policy makers to use AI as a reason to refrain from expanding the radiology workforce or to cut reimbursements for imaging procedures. To reverse the (looming) shortage of radiologists [32, 33], it may be necessary to enroll more residents into radiology training programs, train and employ radiologist assistants [34], and/or increase financial resources to employ (new) radiology staff.

The present study had some limitations. First, the results only apply to the scientific literature published in 2019. Given the lack of previous studies on this topic, the current findings can be considered as a baseline measurement to which future developments can be compared. Second, because of the lack of quantitative data on workload in almost all studies that were included in our analysis, only a qualitative analysis could be done. Third, only the direct impact of each study on workload was assessed. However, scientific innovations and advances in knowledge may allow for more reliable and accurate diagnoses, which may theoretically decrease additional imaging examinations and associated workload. Fourth, besides interpretation time, acquisition and post-processing time were also regarded to have a potential impact on workload, although it can be argued that they may not be the primary tasks of a radiologist. However, the radiologist bears the final responsibility for these processes, and they contribute to the overall workload of a radiology practice. Fifth, the implementation of the findings of the studies that were included in our analysis was based on a theoretical scenario. Whether or not the findings of these studies will eventually be incorporated in guidelines and experience widespread clinical implementation remains unclear. Nevertheless, the results of the present study indicate that there is no reason to assume that workload for radiologists will stabilize let alone decrease. Sixth, our findings apply to both an academic tertiary care center and a non-academic general teaching hospital in the Netherlands, as independently assessed by two radiologists. The quality of healthcare in the Netherlands ranks among the best in Europe [35]. Therefore, the results of this study are likely also applicable to modern radiology departments in other developed countries. However, further research by more radiologists, in other institutions, and in other countries, is necessary to confirm the generalizability of our results.

## Conclusions

In conclusion, recently published medical imaging studies often add value to radiological patient care. However, they likely increase the overall workload of diagnostic radiologists, and this particularly applies to AI studies.

## Supplementary Information

**Additional file 1**: **Appendix 1**. Search term used to retrieve studies from PubMed/Medline that were considered for inclusion. **Appendix 2**. Overview of 440 studies that remained after excluding 560 ineligible studies from the initial sample of 1000 studies.

## Data Availability

All data are available in the manuscript and attached appendices.

## Abbreviation

AI: Artificial intelligence
ESR: European Society of Radiology
OR: Odds ratio
